# Combing machine learning and elemental profiling for geographical authentication of Chinese Geographical Indication (GI) rice

**DOI:** 10.1038/s41538-021-00100-8

**Published:** 2021-07-08

**Authors:** Fei Xu, Fanzhou Kong, Hong Peng, Shuofei Dong, Weiyu Gao, Guangtao Zhang

**Affiliations:** 1 Mars Global Food Safety Center, Beijing, China; 2 Agilent Technologies (China) Co. Ltd., Beijing, China

**Keywords:** Agriculture, Industry, Mass spectrometry

## Abstract

Identification of geographical origin is of great importance for protecting the authenticity of valuable agri-food products with designated origins. In this study, a robust and accurate analytical method that could authenticate the geographical origin of Geographical Indication (GI) products was developed. The method was based on elemental profiling using inductively coupled plasma mass spectrometry (ICP-MS) in combination with machine learning techniques for model building and feature selection. The method successfully predicted and classified six varieties of Chinese GI rice. The elemental profiles of 131 rice samples were determined, and two machine learning algorithms were implemented, support vector machines (SVM) and random forest (RF), together with the feature selection algorithm Relief. Prediction accuracy of 100% was achieved by both Relief-SVM and Relief-RF models, using only four elements (Al, B, Rb, and Na). The methodology and knowledge from this study could be used to develop reliable methods for tracing geographical origins and controlling fraudulent labeling of diverse high-value agri-food products.

## Introduction

Identification of geographical origins of agri-food products is an indispensable first step of the food traceability system, serving as a key to ensuring food quality and safety^1,2^. The concept of geographical indication (GI) first originated during the 19th century in Europe, with the aim of protecting industrial property rights^3^. Nowadays, GI certification has been widely applied to recognize products that possess given quality, reputation, or other characteristics associated with their geographical origins^4^. The European Union enforces the scheme of Protected Geographical Indication as part of its food quality policy, while in China three government sectors supervise and protect different aspects of GIs at the administrative level^5^. These include the State Administration for Industry and Commerce / the Trademark Office, the General Administration of Quality Supervision, Inspection and Quarantine, and the Ministry of Agriculture. Nevertheless, GI products are still frequently mislabeled and adulterated^6,7^ due to the lack of effective analytical methods for ensuring the proper deployment of regulations and monitors^8,9^.

A number of analytical techniques have been proposed for verifying the geographical origins of agri-food products, including elemental profiling^10^, stable isotope analysis^11^, metabolomic fingerprinting^12,13^, and DNA barcoding^14^. Elemental profiling in combination with multivariate analysis (MVA) has attracted the most attention and has been vigorously developed in recent years^15^. The elemental profiles of agri-food products provide valuable evidence of their geographical origins by reflecting topography and soil characteristics^16^. MVA has generally been used to process and integrate large datasets. Principal component analysis (PCA) and discriminant analysis (and its variants) are the two dominant methods in MVA, because of their simplicity in spotting hidden trends embedded in the dataset and their wide availability in commercial analytical software^17^. However, these methods rely on the assumption of a linear relationship between variables to perform well. This could result in inferior prediction performance in real-world scenarios, where sophisticated and nonlinear relationships between predictors are prevalent^18^. In the past decade, an alternative approach using machine learning techniques has proven successful in various research areas^19,20^. These techniques handle large datasets very efficiently and can be implemented easily on open-source platforms^21,22^. Machine learning techniques have superior predictive performance than conventional MVA methods, due to their greater robustness for handling complex relationships within the dataset^23^. Moreover, the reliability and validity of predictive models were significantly improved when they were built with machine learning techniques^24^.

Rice (Oryza sativa L.) is among the world’s three largest food crops and is a staple food for nearly 50% of the world population. China is the leading paddy rice grower globally, producing 220 million metric tons in 2018^25^. Domestic demand for rice with traceable origins is growing with the improvement in living standards. However, Chinese GI rice has become more and more vulnerable to adulteration due to the gap between limited production and high market demand. A scandal in 2010 occurred when ten times more Wuchang rice (a Chinese GI rice) was sold on the market than was produced^26^. Development of a robust and accurate method that can be applied to authenticate the geographical origins of Chinese GI rice will be of great value for protecting the rights and financial interests of farmers, retailers, and consumers.

Elemental profiling has become used more widely to authenticate the geographical origins of premium high-value rice to combat commercial fraud and deliberate mislabeling^27^. However, only a few studies have employed machine learning techniques and no studies have been made in Chinese rice to date. In this study, a new method was developed for tracing the geographical origin of Chinese GI rice using elemental profiling and machine learning techniques. The method could be useful for managing fraudulent labeling of Chinese GI rice in the market, with potential broader application in other GI products.

## Results and discussion

### Concentrations of elements

The measured concentrations of elements in the SRM 1568b agreed well with the certified values (recovery ranged from 80.8% to 102.3%), indicating the high accuracy of the ICP-MS analysis (Table S1). The PCA analysis of the 12 elements measured in both the rice samples and SRM 1568b samples is shown in Fig. S1. The SRM 1568b samples closely clustered together, demonstrating a good reproducibility of analysis. Results from the analysis of the 30 elements in the 131 Chinese GI rice samples are shown in Table S2. Significant differences were observed among all elements across all types of rice (*p* < 0.01), except for Pb (*p* > 0.05). However, these differences were too intricate to clearly indicate which element(s) may contribute the most to the differentiation among six types of GI rice.

### PCA

**Fig. 1:**
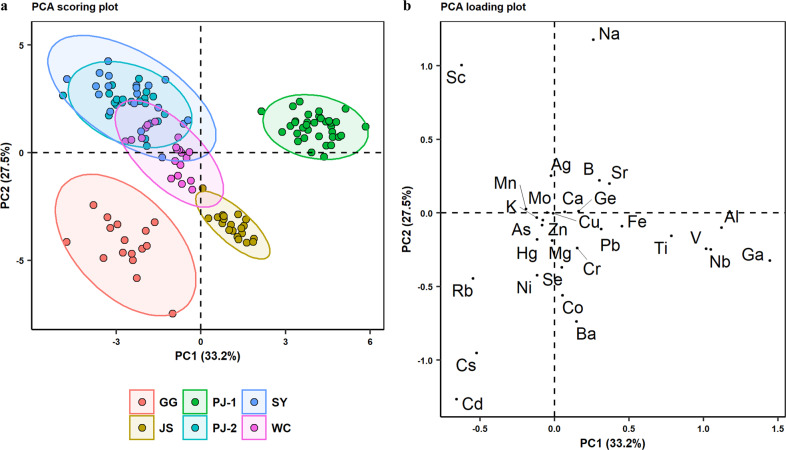
PCA analysis of the 30 elements measured in the 131 Chinese GI rice samples. **a** Scoring plot of PC1 and PC2, with 95% confidence interval eclipse. **b** Loading plot of all features projected on the first two PCs.

The 1st and 2nd principal components (PCs) together accounted for 60.7% of the total variance, and a clear separation was observed among PJ-1, GG, and the other types of GI rice (Fig. 1a). No satisfactory separation was achieved for JS, PJ-2, SY, and WC. The loading plot (Fig. 1b) showed that Al, Ga, Nb, V, and Ti primarily contributed to the variations in PC1, while Na, Sc, Rb, Cs, and Cd contributed to both PC1 and PC2. Notably, PJ-1 and PJ-2 could be clearly separated, despite their common geographical origin (Fig. 1a).

### Identification of geographical origins

High-quality sampling is fundamental for achieving reliable results from multivariate modeling^28^. In this study, we collected all the rice samples from rice processing factories rather than sampling from a market, which ensured the authenticity of samples and minimized the risk of modeling with a “contaminated” dataset. In addition, relatively equal quantities of each variety of rice were collected to provide a balanced dataset, thus preventing the risk of misclassification due to modeling with an imbalanced dataset^20,29^.

**Fig. 2:**
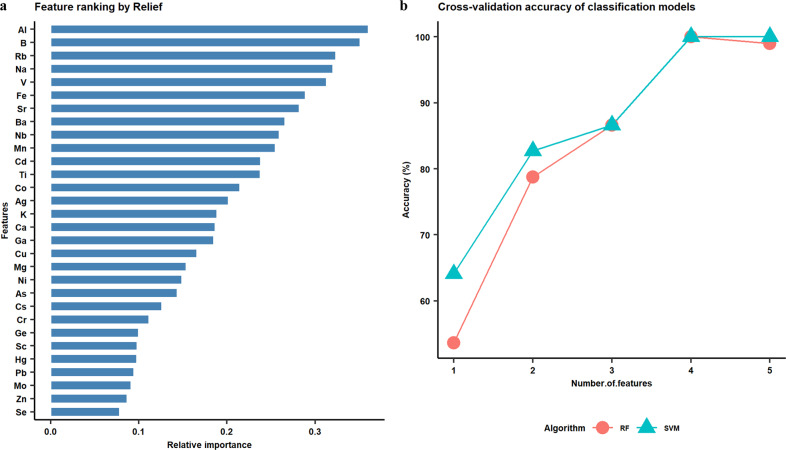
Feature ranking by Relief algorithm and model optimization with cross-validation. **a** Relative importance of each feature based on Relief. **b** Cross-validation accuracy of classification models built with different numbers of features.

Machine learning refers to a collection of algorithms that are capable of constructing prediction models by acquiring and integrating knowledge from large datasets, as well as further improving these models by automatically learning from new knowledge^30^. Machine learning techniques have been applied widely in various research fields, and also show great potential for food traceability^31^. In this study, two widely used supervised classification algorithms, SVM and RF, were implemented for the origin identification of Chinese GI rice based on elemental profiles. In addition, feature selection was applied for model optimization by reducing data dimensions, which is also capable of identifying features with high predictability (also known as biomarkers)^32^. The results of the model training are shown below (Fig. 2 and Fig. S2). The results of feature selection based on the relative importance of each of the 30 elements indicated that Al, B, Rb, Na, and Sr were the main elements that contributed to the differentiation of all types of GI rice (Fig. 2a). This is consistent with the observations in a previous study, where feature selection was also applied and 4 elements (Cd, Rb, Mg, and K) out of 21 evaluated were found to be the most relevant for the differentiation between rice samples from two geographical origins^29^. The performance of both RF and SVM models improved significantly as more features were added, including accuracy (Fig. 2b) and specificity and selectivity (Fig. S2). The mean cross-validation accuracies for RF and SVM were 48% and 63%, respectively with one feature (Al), both reached 100% when four features (Al, B, Rb, and Na) were included. The optimal classifiers were determined as four features with corresponding optimum hyperparameters (max_depth = 26, max_features = ‘auto’, n_estimators = 500 for Relief-RF; ‘linear’ kernel with C value = 1 for Relief-SVM). Feature selection was applied solely to the training set and not to the entire dataset, which eliminated the risk of selection bias^33^.

Cross-validation using a training set can assess the goodness of fit of a particular model. However, independent validation using a separate data set is critical to ultimately evaluate prediction performance, as it incorporates the future working situation^34,35^. Independent validation was conducted in this study using the testing set (Table 1). Both classifiers (Relief-RF and Relief-SVM) could predict the geographical origins of all types of rice with 100% accuracy. The results demonstrated the capability of the machine learning-based method established in this study, especially in constructing reliable predictive models, while simultaneously identifying potential biomarkers accounting for the differentiation.

**Table 1:** Confusion matrix for the independent validation using the testing set.

Classifier	Predicted	Reference						Overall accuracy
		GG (*n* = 4)	JS (*n* = 4)	PJ-1 (*n* = 7)	PJ-2 (*n* = 4)	SY (*n* = 4)	WC (*n* = 4)	
Relief-RF	GG	4	0	0	0	0	0	100%
	JS	0	4	0	0	0	0	
	PJ-1	0	0	7	0	0	0	
	PJ-2	0	0	0	4	0	0	
	SY	0	0	0	0	4	0	
	WC	0	0	0	0	0	4	
Relief-SVM	GG	4	0	0	0	0	0	100%
	JS	0	4	0	0	0	0	
	PJ-1	0	0	7	0	0	0	
	PJ-2	0	0	0	4	0	0	
	SY	0	0	0	0	4	0	
	WC	0	0	0	0	0	4	

### Radar plot analysis

**Fig. 3:**
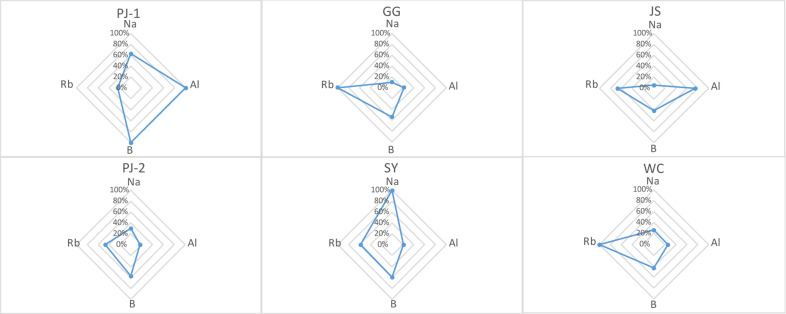
Radar plot of the relative median concentrations for the four features (Al, B, Rb, and Na) in the six types of Chinese GI rice. The graph displays differences in elemental patterns among geographical origins. Each subgraph corresponds to a different GI variety.

The differentiation power of the four features is visualized in the plot of relative median concentrations (Fig. 3). The elemental profile of each GI rice was significantly different. It is noteworthy that the concentration of Al was highest in PJ-1 and lowest in PJ-2, even though PJ-1 and PJ-2 were sampled from the same geographical location. In addition, the other three elements were present in considerably different proportions in PJ-1 and PJ-2. These observations agreed with previous findings that cultivar types also play a significant role in the composition of elements in rice kernels^36,37^. The significant difference of Al concentrations between PJ-1 and PJ-2 indicated that the genotype of rice could contribute more to the variation of Al in rice, comparing with geographic region. It remains a challenge to elucidate why the four elements showed such strong differentiation power, as the sample set used in this study was diverse and complex. The samples were from the three dominant rice-producing regions of China, including the northeast China plain (WC, PJ-1, and PJ-2), Yangtze River Basin (SY, JS), and southeast coastal region (GG). Such geographically wide sampling introduced multiple variables, including soil characteristics, agricultural practices, and genotype variations, all of which could affect the elemental profile of crops^38,39^. Similar findings have been reported by Qian et al.^40^ in a study on the determination of the geographical origin of Wuchang rice (one type of Chinese GI rice) using elemental profiling. Likewise, elements of Na, Al, and Rb were identified with significant differences among various geographic origins and were applied to establish the discrimination model where all the Wuchang rice samples were successfully separated from the other rice samples. Moreover, the genotype variation was also demonstrated as Cu showed significant differences among samples of different genotypes.

A study on Brazilian rice^29^ reported that Cd only could differentiate rice samples from two geographical origins, and it was the difference in irrigation methods that resulted in the variance of Cd content. Cadmium was detected in all six types of Chinese GI rice, with the highest concentration in GG from Guangxi province (Table S2). A previous national-scale study reported that the concentrations of Cd in paddy soils varied significantly in different regions of China, and were higher in southeast coastal regions such as Guangxi province^41^. The feasibility of using only Cd to differentiate between GG and non-GG rice samples was also evaluated in this study. The original dataset was regrouped as GG and non-GG, and the developed machine learning-based workflow was applied. The result of feature selection indicated that Cd was the element with the highest relative importance, and the prediction accuracy of 100% was achieved using Cd alone, by both Relief-SVM and Relief-RF models. These results again demonstrated the effectiveness of the developed machine learning-based method, which is valid not only for the discrimination of multiple varieties but also for the differentiation of relatively fewer varieties using the least number of features, with the potential of greatly improving working efficiency and productivity.

In conclusion, a reliable method for tracing the geographical origins of Chinese GI rice was successfully developed using machine learning models built with multielement fingerprints. A series of predictive models were established, serving various purposes. Two predictive models were established for the classification of six GI varieties simultaneously, and 100% prediction accuracy was achieved with a feature set of four elements. Another set of models successfully discriminated one GI variety from others, with Cd identified as the predictor with the most discriminatory power. A comprehensive workflow for machine learning modeling has been provided and all important factors for building reliable classification models have been discussed. This method provides a basis for others to develop fit-for-purpose methods for tracing origins of other valuable agri-food products with designated origins, as well as discovering key elemental biomarkers associated with their geographical locations.

## Methods

### Sample collection

One hundred and thirty-one Chinese GI rice samples with six GI varieties were directly collected from rice processing factories in five provinces in China, including Heilongjiang and Liaoning [two sample sets] in the northeastern production area, Jiangsu in the eastern production area, Hubei in the mid-southern production area and Guangxi in the southeastern production area. These are labeled as WC, PJ-1, PJ-2, SY, JS, and GG, respectively, in the remainder of this manuscript. Sample numbers obtained from each region are as follows: WC (*n* = 20), PJ-1 (*n* = 35), PJ-2 (*n* = 20), SY (*n* = 20), JS (*n* = 20), and GG (*n* = 16).

### Reagents and standards

Nitric acid (69%, part# 100441) was purchased from Merck Millipore (Darmstadt, Ger-many). Deionized water (DIW, 18.2MΩ·cm) was obtained from a Milli-Q system (Millipore, MA, USA). Multi-element calibration standard 2A (part# 8500-6940) and 4 (part# 8500-6942), environmental calibration standard (part# 5183-4688), and standard solutions of scandium (Sc, part# 5190-8578) and rhodium (Rh, part# 8500-6945) were purchased from Agilent Technologies (Santa Clara, CA, USA). The Standard Reference Material (SRM) of rice flour (1568b) was purchased from the National Institute of Standards and Technology (NIST, Gaithersburg, MD, USA).

### ICP-MS analysis

Rice samples were pre-processed and acid digested according to the method recently published^42^. A portion of 0.5 g of rice grains was weighed in a polytetrafluoroethylene (PTFE) digestion vessel and mixed with 6 mL of nitric acid. The vessel was placed in a fume hood overnight for pre-digestion and then transferred into the microwave oven (Anton Paar, Austria) for acid digestion. The digestion temperature of 180 °C was gradually reached in 15 min and held for 20 min. Then the solution was cooled to room temperature and diluted to 50 mL with DIW. Before usage, all materials including the digestion vessels were soaked in a 30% (*v*/*v*) nitric acid solution for 24 h and rinsed with DIW three times to avoid cross-contamination.

The concentrations of 30 elements (B, Na, Mg, Al, K, Ca, Sc, Ti, V, Cr, Mn, Fe, Co, Ni, Cu, Zn, Ga, Ge, As, Se, Rb, Sr, Nb, Mo, Ag, Cd, Cs, Ba, Hg, and Pb) were measured using an Agilent 7900 ICP-MS (Agilent Technologies, Santa Clara, CA, USA). The instrumental setting and operating conditions were adopted from a previously published method^43^ with some modifications. In brief, only helium tune mode was used, and the plasma parameters were as follows: radio frequency power 1550 W; sampling depth 8 mm; carrier gas flow (Argon) 1.16 L·min^−1^; cell gas (helium) flow 5.0 mL·min^−1^. The calibration solution was prepared by mixing and diluting the standards mentioned in the previous section (except for Rh). A diluted Rh standard solution (1 mg·L^−1^) was used as the internal standard to correct matrix effects and to compensate for possible instrument deviations. It was mixed with the sample stream using a tee joint. The accuracy and reproducibility of analysis were verified by analyzing the rice flour SRM 1568b once every ten samples. Each rice sample was analyzed in duplicate.

### Statistical analysis

One-way analysis of variance (ANOVA) coupled with Tukey’s test (*p* < 0.05) was used for preliminary analysis of the concentration of the 30 elements in each GI rice. The dataset was then scaled through logarithmic transformation and subjected to unsupervised PCA for the initial visualization of data distribution. Subsequently, the dataset was used to construct predictive models with machine learning algorithms.

### Machine learning modeling

**Fig. 4:**
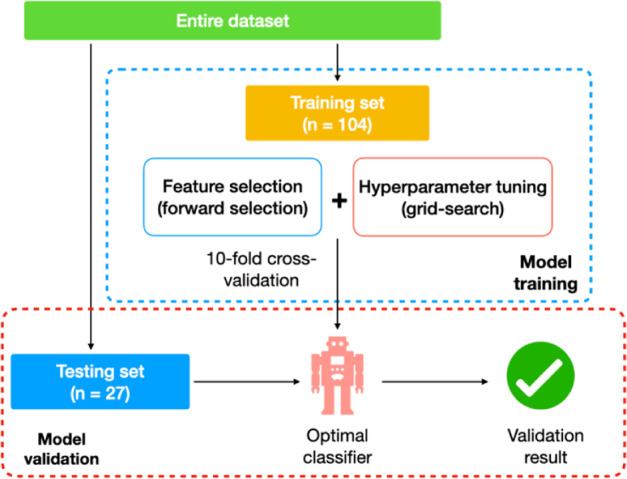
Diagram of the proposed machine learning-based workflow. The flowchart describes the entire process of the developed machine learning-based data processing workflow, including all important factors of the data partition, feature selection, hyperparameter tuning, and model validation. The steps for model training and model validation are outlined in boxes with blue and red dashed lines, respectively.

Two machine learning algorithms, RF and SVM, were implemented to construct predictive models. RF is an ensemble of decision trees that are generated from the original dataset using bootstrap partition^44^. SVM implements classifications by projecting input vectors into a high dimensional space, thus finding a hyperplane that could separate different classes^45^. Feature selection is a data mining technique, aiming to identify pertinent features, as well as optimize predictive models, through discarding irrelevant ones that are not informative but contribute to the overall dimensionality of the problem space^46^. In our study, the Relief algorithm was utilized to select features through investigating their relative importance based on a calculated proxy statistic^47^. Specifically, we have proposed a machine learning-based workflow for unbiased feature selection, model construction, and performance evaluation (below and Fig. 4).

1. The scaled dataset was randomly split into a training set (*n* = 104) and a testing set (*n* = 27) in a stratified fashion (80:20).
2. Feature selection was applied to the training set and all the 30 features were ranked based on their differentiation power. Subsequently, stepwise forward selection^48^ was conducted along with hyperparameter tuning (grid-search). After 10-fold cross-validation, the best combinations of feature subsets and hyperparameters were used to construct optimal classifiers. The tested hyperparameters can be found in Table S3.
3. The optimal classifiers were then independently validated on the testing set, and their prediction accuracies were determined.

All statistical analyses and model development were carried out on R version 3.5.1 (packages factoextra^49^, tidyverse^50^, and agricolae^51^) and Python version 3.7 (packages skearn^52^ and skrebate^53^).

### Reporting summary

Further information on research design is available in the Nature Research Reporting Summary linked to this article.

## Supplementary information

Supplementary Information

Reporting Summary

## Data Availability

The authors declare that all relevant data supporting this study has been included in the paper and supplementary materials; raw data will be available from the corresponding author upon reasonable request.

## Code availability

The source code used in this manuscript can be found in the GitHub repository upon request: https://github.com/lancelot0821/Special_issue_rice_authenticity.
