# Supplementary material for: Combing machine learning and elemental profiling for geographical authentication of Chinese Geographical Indication (GI) rice
Source: NPJ Sci Food. 2021 Jul 8;5:18. doi: 10.1038/s41538-021-00100-8 (PMC8266907; doi:10.1038/s41538-021-00100-8)
Supplement: Supplementary file 1 — Supplementary Information [file 41538_2021_100_MOESM1_ESM.pdf]

Supplementary material

Complete tables of recoveries of elements in the standard reference material (SRM) 1568b, concentrations of the 30 elements in the 131 Chinese GI rice samples, and ranges of tested model hyperparameters. Figures of PCA plot of the elements measured in both the rice samples and SRM samples and statistics for model training with 10-fold cross-validation are also provided.

Table S1. Recoveries of elements in the SRM 1568b

Element	Certified value (mg/kg)	Measured value (mg/kg)	Recovery (%)
Mg	559 ± 10	526 ± 27	94.7
Al	4.21 ± 0.34	4.08 ± 0.14	97.4
K	1282 ± 11	1281 ± 58	99.2
Ca	118.4 ± 3.1	117.8 ± 7.0	101.9
Mn	19.2 ± 1.8	17.5 ± 5.9	91.8
Fe	7.42 ± 0.44	7.15 ± 0.41	98.6
Cu	2.35 ± 0.16	2.18 ± 0.18	95.8
Zn	19.42 ± 0.26	16.34 ± 0.76	85.5
As	0.285 ± 0.014	0.282 ± 0.024	102.3
Se	0.365 ± 0.029	0.348 ± 0.051	95.5
Mo	1.451 ± 0.048	1.272 ± 0.074	89.7
Cd	0.0224 ± 0.0013	0.0180 ± 0.0010	80.8

Table S2. Concentrations of the 30 elements in the 131 Chinese GI rice samples

	Type of Chinese GI Rice						
Element	GG (n=16)	JS (n=20)	PJ-1（n=35）	PJ-2 (n=20)	WC (n=20)	SY (n=20)	*p* value
B (μg/kg)	392.50 ± 41.03[b]	302.00 ± 36.18[c]	727.39 ± 75.75[a]	425.56 ± 49.09[b]	320.33 ± 28.38[c]	440.56 ± 50.61[b]	7.54E-62
Na (mg/kg)	2.08 ± 0.19[de]	1.18 ± 0.12[e]	14.25 ± 6.41[b]	6.00 ± 0.63[c]	5.30 ± 0.59[cd]	20.00 ± 3.14[a]	2.60E-39
Mg (mg/kg)	257.01 ± 20.61[a]	254.94 ± 16.05[a]	187.29 ± 22.75[b]	187.41 ± 13.29[b]	183.24 ± 13.86[b]	185.46 ± 22.02[b]	7.00E-34
Al (mg/kg)	0.40 ± 0.08[c]	1.48 ± 0.21[b]	2.36 ± 0.79[a]	0.33 ± 0.06[c]	0.53 ± 0.13[c]	0.40 ± 0.08[c]	1.75E-42
K (mg/kg)	961.45 ± 65.69[b]	770.48 ± 44.69[d]	723.98 ± 75.70[de]	702.17 ± 45.36[e]	842.62 ± 45.82[c]	1053.24 ± 104.88[a]	2.46E-38
Ca (mg/kg)	63.14 ± 6.30[a]	43.81 ± 2.36[c]	63.56 ± 7.03[a]	55.93 ± 3.27[b]	56.81 ± 3.34[b]	46.44 ± 3.57[c]	1.27E-31
Sc (μg/kg)	0.07 ± 0.08[bc]	0.10 ± 0.07[b]	0.23 ± 0.16[a]	0.01 ± 0.02[c]	0.07 ± 0.08[bc]	0.02 ± 0.03[bc]	3.01E-14
Ti (μg/kg)	38.74 ± 19.18[bc]	48.22 ± 11.75[b]	89.01 ± 26.74[a]	19.67 ± 6.75[d]	31.07 ± 8.17[cd]	23.68 ± 8.12[cd]	1.67E-32
V (μg/kg)	1.01 ± 0.26[cd]	2.58 ± 0.33[b]	3.71 ± 0.75[a]	0.54 ± 0.12[e]	1.19 ± 0.30[c]	0.71 ± 0.19[de]	1.14E-58
Cr (μg/kg)	15.97 ± 6.26[b]	33.49 ± 37.03[a]	15.73 ± 7.38[b]	11.48 ± 2.64[b]	10.89 ± 3.75[b]	16.75 ± 5.28[b]	1.20E-4
Mn (μg/kg)	9.91 ± 0.99[b]	5.15 ± 0.49[e]	7.03 ± 0.80[d]	8.71 ± 0.75[c]	13.53 ± 0.85[a]	7.45 ± 0.68[d]	9.04E-66
Fe (mg/kg)	2.66 ± 0.57[b]	2.62 ± 0.42[b]	4.06 ± 0.85[a]	1.53 ± 0.12[d]	1.53 ± 0.13[d]	2.05 ± 0.23[c]	1.66E-39
Co (μg/kg)	8.09 ± 1.15[a]	5.24 ± 0.66[b]	3.26 ± 0.49[c]	2.01 ± 0.30[e]	2.81 ± 0.75[cd]	2.53 ± 0.42[de]	3.59E-59
Ni (μg/kg)	243.99 ± 57.89[a]	190.93 ± 28.04[b]	101.99 ± 21.33[d]	83.60 ± 21.27[d]	130.23 ± 36.40[c]	148.01 ± 29.83[c]	4.15E-32
Cu (mg/kg)	2.42 ± 0.30[a]	1.83 ± 0.14[c]	2.13 ± 0.24[b]	1.74 ± 0.15[c]	1.71 ± 0.14[c]	2.57 ± 0.24[a]	4.57E-30
Zn (mg/kg)	14.66 ± 1.29[a]	11.12 ± 0.64[bc]	10.58 ± 0.98[c]	11.57 ± 0.86[b]	11.23 ± 0.59[bc]	11.49 ± 0.81[b]	4.38E-27
Ga (μg/kg)	0.22 ± 0.08[c]	0.56 ± 0.15[b]	1.20 ± 0.60[a]	0.10 ± 0.05[c]	0.31 ± 0.12[bc]	0.11 ± 0.08[c]	4.38E-27
Ge (μg/kg)	1.59 ± 0.22[b]	1.67 ± 0.23[b]	2.11 ± 0.41[a]	2.14 ± 0.44[a]	1.56 ± 0.29[b]	1.15 ± 0.25[c]	1.23E-18
As (μg/kg)	103.78 ± 12.31[b]	110.52 ± 8.72[b]	90.78 ± 7.79[c]	125.56 ± 16.05[a]	114.63 ± 18.40[ab]	88.45 ± 11.13[c]	3.28E-19
Se (μg/kg)	48.60 ± 12.10[a]	53.51 ± 17.15[a]	32.01 ± 9.86[b]	34.20 ± 9.90[b]	28.09 ± 9.17[bc]	20.32 ± 9.24[c]	3.86E-16
Rb (mg/kg)	2.22 ± 0.54[a]	1.42 ± 0.11[b]	0.53 ± 0.09[d]	1.02 ± 0.18[c]	2.14 ± 0.17[a]	1.21 ± 0.30[bc]	1.81E-51
Sr (μg/kg)	67.66 ± 13.59[cd]	57.78 ± 5.18[d]	145.15 ± 23.96[a]	97.90 ± 6.72[b]	75.87 ± 6.75[c]	56.64 ± 5.37[d]	4.84E-52
Nb (μg/kg)	0.08 ± 0.04[c]	0.13 ± 0.03[b]	0.24 ± 0.07[a]	0.03 ± 0.01[c]	0.06 ± 0.02[c]	0.04 ± 0.01[c]	1.40E-38
Mo (μg/kg)	445.78 ± 71.50[a]	318.88 ± 32.38[d]	378.99 ± 34.02[bc]	344.26 ± 28.46[cd]	412.87 ± 59.05[ab]	427.95 ± 40.65[a]	6.68E-16
Ag (μg/kg)	2.24 ± 0.36[a]	0.48 ± 0.14[d]	1.91 ± 0.39[b]	1.29 ± 0.26[c]	1.39 ± 0.23[c]	1.78 ± 0.24[b]	3.45E-38

Cd (µg/kg)	228.36 ± 37.34^{a}	43.63 ± 11.93^{b}	9.03 ± 3.95^{c}	10.53 ± 3.82^{c}	16.70 ± 6.91^{c}	18.68 ± 4.85^{c}	1.10E-84
Cs (µg/kg)	18.76 ± 22.87^{a}	1.68 ± 0.18^{b}	0.83 ± 0.15^{b}	1.06 ± 0.23^{b}	2.05 ± 0.35^{b}	1.07 ± 0.28^{b}	4.07E-10
Ba (µg/kg)	181.91 ± 26.94^{a}	158.70 ± 17.87^{b}	74.31 ± 34.07^{c}	73.29 ± 10.79^{cd}	54.73 ± 10.15^{d}	25.10 ± 4.44^{e}	2.49E-49
Hg (µg/kg)	3.24 ± 1.00^{a}	1.32 ± 0.23^{c}	1.64 ± 0.30^{c}	1.48 ± 0.36^{c}	2.23 ± 0.86^{b}	1.64 ± 0.42^{c}	1.55E-18
Pb (µg/kg)	15.02 ± 9.85^{a}	10.61 ± 7.98^{a}	15.83 ± 8.60^{a}	10.93 ± 9.37^{a}	8.23 ± 12.03^{a}	11.52 ± 10.91^{a}	0.084

Note: ^{abcde} Significant difference at *p*=0.05 confidence level.

Table S3. Ranges of hyperparameters tested during model training

Hyperparameters	RF	SVM
max_depth	[1, 26, 51, 76, 101]	NA
max_features	['auto', 'sqrt']	NA
n_estimators	[100, 200, 300, 400, 500]	NA
Cost	NA	[0.1, 1, 10, 100, 1000]

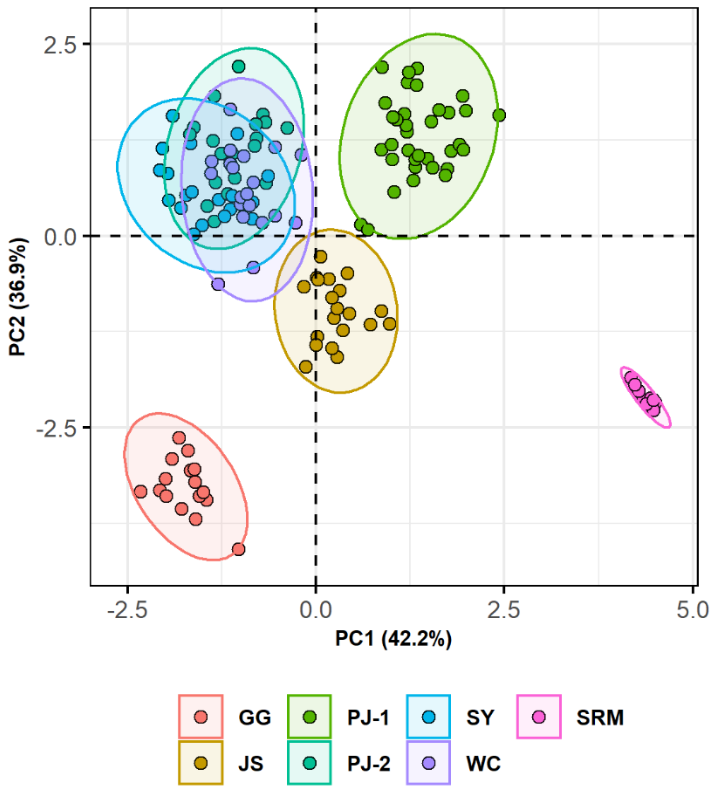

Figure S1: PCA analysis of the 12 elements measured in both the 131 Chinese GI rice samples and the NIST SRM 1568b samples. Scoring plot of PC1 and PC2, with 95% confidence interval eclipse.

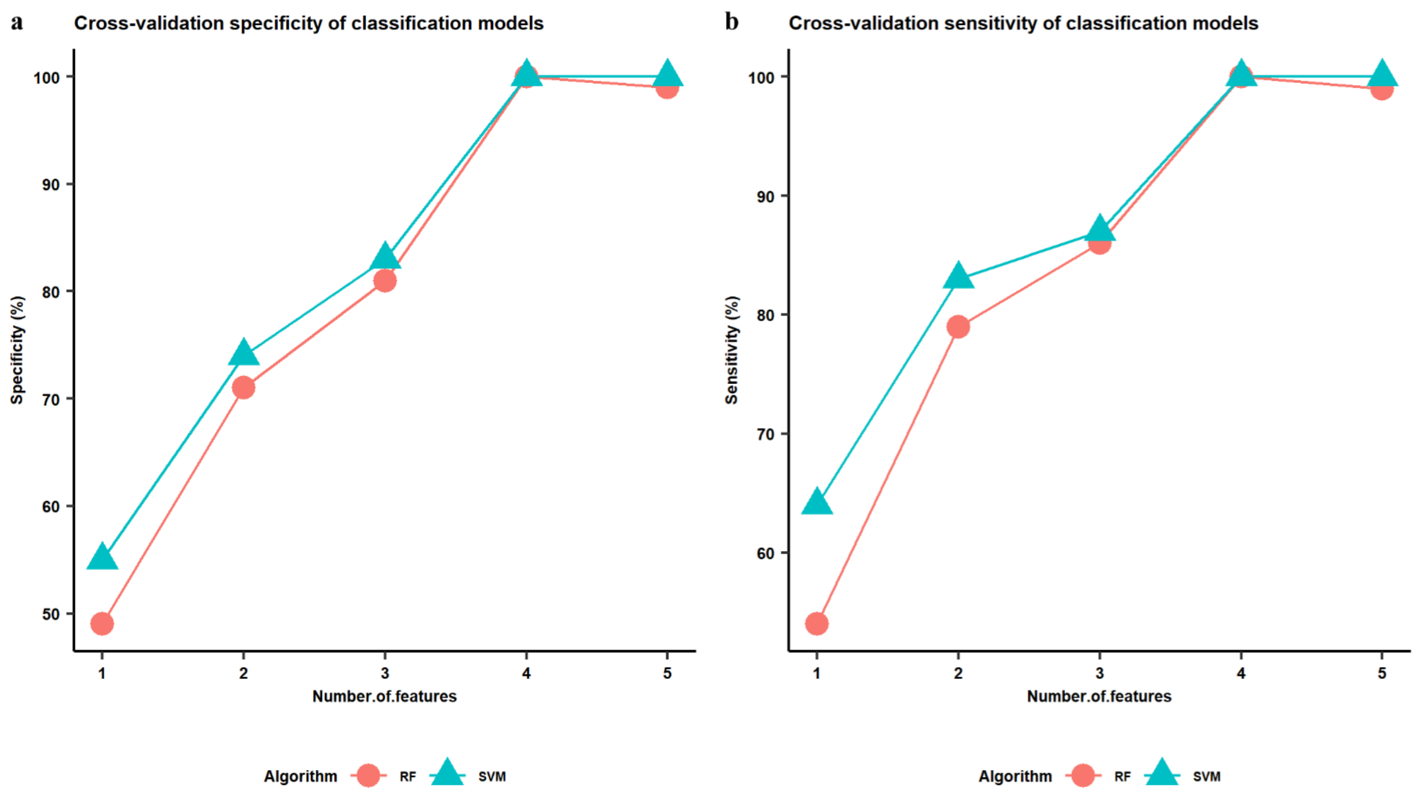

Figure S2: Additional statistics of model optimization with cross-validation. a Cross-validation specificity of classification models built with different numbers of features. **b** Cross-validation sensitivity of classification models built with different numbers of features.
