# Supplementary material for: Combing machine learning and elemental profiling for geographical authentication of Chinese Geographical Indication (GI) rice
Source: NPJ Sci Food. 2021 Jul 8;5:18. doi: 10.1038/s41538-021-00100-8 (PMC8266907; doi:10.1038/s41538-021-00100-8)
Supplement: Supplementary file 2 — Reporting Summary [file 41538_2021_100_MOESM2_ESM.pdf]

nature research

Corresponding author(s): Hong Peng: hong.peng@effem.com

Last updated by author(s): May 8, 2021

Reporting Summary

Nature Research wishes to improve the reproducibility of the work that we publish. This form provides structure for consistency and transparency in reporting. For further information on Nature Research policies, see our Editorial Policies and the Editorial Policy Checklist.

Statistics

For all statistical analyses, confirm that the following items are present in the figure legend, table legend, main text, or Methods section.

n/a	Confirmed	
☐	☒	The exact sample size (*n*) for each experimental group/condition, given as a discrete number and unit of measurement
☐	☒	A statement on whether measurements were taken from distinct samples or whether the same sample was measured repeatedly
☐	☒	The statistical test(s) used AND whether they are one- or two-sided<br>*Only common tests should be described solely by name; describe more complex techniques in the Methods section.*
☒	☐	A description of all covariates tested
☒	☐	A description of any assumptions or corrections, such as tests of normality and adjustment for multiple comparisons
☐	☒	A full description of the statistical parameters including central tendency (e.g. means) or other basic estimates (e.g. regression coefficient) AND variation (e.g. standard deviation) or associated estimates of uncertainty (e.g. confidence intervals)
☐	☒	For null hypothesis testing, the test statistic (e.g. *F*, *t*, *r*) with confidence intervals, effect sizes, degrees of freedom and *P* value noted<br>*Give P values as exact values whenever suitable.*
☒	☐	For Bayesian analysis, information on the choice of priors and Markov chain Monte Carlo settings
☒	☐	For hierarchical and complex designs, identification of the appropriate level for tests and full reporting of outcomes
☒	☐	Estimates of effect sizes (e.g. Cohen's *d*, Pearson's *r*), indicating how they were calculated

Our web collection on statistics for biologists contains articles on many of the points above.

Software and code

Policy information about availability of computer code

Data collection	The data was collected with Agilent MassHunter version 4.4.
Data analysis	ANOVA and PCA were implemented with RStudio 3.5.1. Feature selection, model construction and validation were performed with Python 3.7.

For manuscripts utilizing custom algorithms or software that are central to the research but not yet described in published literature, software must be made available to editors and reviewers. We strongly encourage code deposition in a community repository (e.g. GitHub). See the Nature Research guidelines for submitting code & software for further information.

Data

Policy information about availability of data

All manuscripts must include a data availability statement. This statement should provide the following information, where applicable:

- Accession codes, unique identifiers, or web links for publicly available datasets
- A list of figures that have associated raw data
- A description of any restrictions on data availability

The source code used in this manuscript can be found in the GitHub repository: https://github.com/lancelot0821/Special_issue_rice_authenticity.

Field-specific reporting

Life sciences study design

All studies must disclose on these points even when the disclosure is negative.

Sample size	No sample-size calculation was performed. The sample size of each rice variety is comparable to the sample size reported in literature. And a relatively equal sample size of each rice variety was collected to provide a balanced sample set.
Data exclusions	No data was excluded from the analysis.
Replication	The reproducibility was evaluated through measuring the recoveries of elements in standard reference materials (SRMs) during analysis. The SRM samples were analyzed 10 times in total and all the replications were successful.
Randomization	Randomization is not applicable to this study since samples were not allocated into experimental groups.
Blinding	Blinding is not applicable to this study since no individual was involved in group allocation.

Reporting for specific materials, systems and methods

We require information from authors about some types of materials, experimental systems and methods used in many studies. Here, indicate whether each material, system or method listed is relevant to your study. If you are not sure if a list item applies to your research, read the appropriate section before selecting a response.

Materials & experimental systems

n/a	Involved in the study	
☒	☐	Antibodies
☒	☐	Eukaryotic cell lines
☒	☐	Palaeontology and archaeology
☒	☐	Animals and other organisms
☒	☐	Human research participants
☒	☐	Clinical data
☒	☐	Dual use research of concern

Methods

n/a	Involved in the study	
☒	☐	ChIP-seq
☒	☐	Flow cytometry
☒	☐	MRI-based neuroimaging
